# Case of Cerebro-Spinal Meningitis Successfully Treated

**Published:** 1871-09

**Authors:** Wm. O’Daniel

**Affiliations:** Twiggs County, Georgia


### CASE OF CEREBRO-SPINAL MENINGITIS SUCCESSFULLY TREATED.

By Wm. O'Daniel, M.D., Twiggs County, Georgia.

On the 7th of July last, at four o'clock p.m., I visited a very stout young negro woman, who possessed very great muscular power and robust health up to a few weeks previously to the time above referred to, when she had several attacks of intermittent fever, which, I was informed, did not yield very readily to treatment resorted to by her friends, and that the chills returned on the seventh, fourteenth and twenty-first days, as they often do in this malarious section of country, though she had enjoyed good health for several days previously to the attack which I diagnosticated as above.

Upon inquiry, I found that her monthly visitations had always been regular, and that she had always enjoyed fine health until the last few weeks, as stated above. Had never borne children. Had always lived in a section of country free from malarial influence, until the past year.

Upon a close examination, I found the following symptoms existing: Opisthotonos, delirious, eyes red, pupils dilated, tongue natural, bowels constipated, pulse one hundred and fifteen, and very feeble, respiration hurried, breathing stertorous, perspiring freely, urine natural, both in color and quantity. She was perfectly insensible, and said to have suffered a most excruciating pain in the head before she became entirely insensible.

By the time I had made my prescription, I discovered tetanic symptoms, with increased opisthotonos. I at once resorted to copious bleeding from the arm. I then attempted to give her hyd. chlor. mit., grs. xx, and found deglutition impossible. I then ordered an enema of strong salt water, which, I presume, together with relaxation caused from phlebotomy, produced a fine evacuation from the bowels. At this time, she being very restless, I gave her one grain of morphine hypodermically over left orbital ridge; applied blister from occiput to first dorsal vertebræ, and frictions of oil of turpentine over the full length of spine not covered with blister, and left, requesting her friends to notify me if she lived through the night.

I visited her at 8 o'clock on the morning of the 8th of July. I found her resting quietly, opisthotonos continuing, breathing more natural, pulse ninety per minute, and still insensible, but could, with difficulty, be induced to swallow. I then ordered hyd. chlor. mit. and sulph. quinine, aa grs. xx., m. ft. chart. three; one to be taken every two hours, and one-half grain of morphine at nine o'clock P.M.; cold applications to head until my return.

Visited patient again on the morning of the 9th, and found her able to speak rationally; took nourishment; medicines given day before had the desired effect. Left fifteen grains of quinine, to be given in five-grain doses every two hours, and morphine to be given in one-fourth grain doses as occasion may require to promote rest, and dismissed the case.

The woman continued to improve, and in a few days was able to labor on the farm. Have seen her since, and found no traces of disease.

I have had a good many similar cases the last few years, and always treat them according to circumstances, as I don't know that I have ever found two exactly alike—not adopting, therefore, any particular plan of treatment; this, however, was one of the few cases in which I have thought proper to employ blood-letting. I have often had the pleasure of my friend Dr. S. L. Richardson's views in regard to such cases, who is an eminent medical gentleman, and whose experience is worthy of attention.

I must say, though I am well aware that some of the ablest medical gentlemen in the State differ from me, that, in my opinion, cerebro-spinal meningitis is generally caused from malarial influence, as I have mostly found the disease where fevers of the intermittent and remittent types prevail. Dr. Richardson concurs with me in this opinion.

